# Dr. Frederick J. Bancroft

**Published:** 1903-02

**Authors:** 


Dr. Frederick J. Bancroft, of Denver, Col., died at San Diego, Cal., January 16, 1903, of disease of the heart, aged 68 years. He graduated in medicine at the University of Buffalo in 1861. He served as a surgeon during the civil war, after which he located at Denver in 1866, and became one of the most prominent physicians in that state.

Dr. Bancroft served as president of a number of medical organisations and was the first president of the Colorado State Board of Health.

